# Investigate the relationship between the retraction reasons and the quality of methodology in non-Cochrane retracted systematic reviews: a systematic review

**DOI:** 10.1186/s13643-023-02439-3

**Published:** 2024-01-12

**Authors:** Azita Shahraki-Mohammadi, Leila Keikha, Razieh Zahedi

**Affiliations:** 1 Medical Library and Information Sciences, School of Allied Medical Sciences, Zahedan University of Medical Sciences, Zahedan, Iran; 2 Medical Librarianship and Information Sciences, Tehran University of Medical Sciences, Tehran, Iran

**Keywords:** Retraction, Systematic reviews, Methodology, Quality, AMSTAR-2

## Abstract

**Background:**

This systematic review aimed to investigate the relationship between retraction status and the methodology quality in the retracted non-Cochrane systematic review.

**Method:**

PubMed, Web of Science, and Scopus databases were searched with keywords including systematic review, meta-analysis, and retraction or retracted as a type of publication until September 2023. There were no time or language restrictions. Non-Cochrane medical systematic review studies that were retracted were included in the present study. The data related to the retraction status of the articles were extracted from the retraction notice and Retraction Watch, and the quality of the methodology was evaluated with the AMSTAR-2 checklist by two independent researchers. Data were analyzed in the Excel 2019 and SPSS 21 software.

**Result:**

Of the 282 systematic reviews, the corresponding authors of 208 (73.75%) articles were from China. The average interval between publish and retraction of the article was about 23 months and about half of the non-Cochrane systematic reviews were retracted in the last 4 years. The most common reasons for retractions were fake peer reviews and unreliable data, respectively. Editors and publishers were the most retractors or requestors for retractions. More than 86% of the retracted non-Cochrane SRs were published in journals with an impact factor above two and had a critically low quality. Items 7, 9, and 13 among the critical items of the AMSTAR-2 checklist received the lowest scores.

**Discussion and conclusion:**

There was a significant relationship between the reasons of retraction and the quality of the methodology (*P*-value < 0.05). Plagiarism software and using the Cope guidelines may decrease the time of retraction. In some countries, strict rules for promoting researchers increase the risk of misconduct. To avoid scientific errors and improve the quality of systematic reviews/meta-analyses (SRs/MAs), it is better to create protocol registration and retraction guidelines in each journal for SRs/MAs.

**Supplementary Information:**

The online version contains supplementary material available at 10.1186/s13643-023-02439-3.

## Introduction

A systematic review (SR) is a scientific investigation that focuses on a specific question and uses explicit, pre-specified scientific methods to identify, select, assess, and summarize the findings of similar but separate studies [1]. The meta-analysis (MA) is a statistical method that aims to pool individual results from homogeneous primary studies quantitatively, and SRs are often but not always accompanied by MAs [2]. SRs are widely used in many fields of medicine, especially in evidence-based practice [3, 4], and are at the highest level of the pyramid of studies for evidence-based decision-making [5]. SRs/MAs summarize and synthesize data to advance future research and evidence-based practice [6] and they are used as the most important evidence to create clinical guidelines [7, 8].

According to the Committee on Publication Ethics (COPE), retraction of articles is defined as a process for correcting scientific studies and informing readers whose publication may contain incomplete or erroneous data [9]. In the guidelines of COPE, honest mistakes, plagiarism, author issues, overlapping, and duplicate publications are mentioned as reasons for the retraction of articles [9]. Investigations show that the reasons for the retraction of SRs/MAs include errors, plagiarism, scientific misconduct, or mistakes that reduce the scientific validity of published results and lead to the official cancellation of publications, known as retraction [10, 11].

Evidence shows that the number of retracted articles, especially in medicine, has expanded in recent decades [12]. Recently, retracting articles due to scientific misconduct has attracted more attention in scientific societies [12, 13]. In a study of retracted biomedical articles from 1971 to 2020, SRs/MAs studies ranked second among the retracted studies [13]. In addition, the retraction of a significant number of SRs by Chinese authors has attracted a lot of attention due to fake peer reviews [14, 15]. In addition to the reasons for retraction provided by COPE, in terms of methodology, the selection of appropriate keywords and comprehensive search are essential in SRs, because misinterpretation of the results can affect the quality of medical procedures [16] or the health of patients [17]; the quality and methodological accuracy of SR articles can be compromised as well.

While SRs/MAs should be based on a comprehensive evaluation of the available evidence, in some cases, incomplete reporting of the results was observed [18]. Failure to use a standard guideline to report studies SRs/MAs leads to uncertain results and eventually incorrect guidance in evidence-based practice [19]. AMSTAR-2 was developed as a critical appraisal tool for SR/MA that includes randomized or non-randomized studies of health care interventions, or both of them but does not include other types of study such as diagnostic test accuracy, individual patient data, or scoping review. AMSTAR-2 contains 16 questions, and four options could be used to answer these questions: yes, partial yes, No, or No meta-analysis conducted [20]. Although AMSTAR 2 is not intended to generate an overall score by calculating the critical items, including Items 2, 4, 7, 9, 11, 13, and 15 the final decision is possible about the quality of the methodology of the articles [20]. Considering the importance of SRs/MAs in the medical sciences, this study was done to investigate: (1) descriptive characteristics and reasons for retraction, (2) assess the quality of methodology, and (3) analyze the relationship between methodological quality and reasons for retraction.

## Methodology

This study is not registered and is reported based on the Preferred Reporting Items of Systematic Reviews and Meta-Analysis (PRISMA) checklist.

### Search strategy

Three databases including PubMed, Scopus, and Web of Science (WOS) were searched until September 2023 to collect retracted non-Cochrane SRs. For example, in the PubMed database, articles were extracted by searching the keyword including the retracted publication [sb] and using meta-analysis and systematic review filters. Detailed search strategy is provided in Additional file 1. The data related to the retraction reasons of the reviewed articles were extracted from the interpretation of the retraction notice of the articles and the Retraction Watch database which provides more details of the retraction reasons for each article.

Website address: (http://retractiondatabase.org/RetractionSearch.aspx?).

### Inclusion and exclusion criteria

The inclusion criteria were retracted for non-Cochrane SRs in the medical field and no language and time restrictions were applied. The articles were excluded for reasons including no systematic review and NRSI, non-interventional studies including etiology/diagnosis/epidemiology/prognosis systematic review, and a network meta-analysis, design system, or scoping review.

### Study selection

Two reviewers (ASM and LK) independently screened the titles and abstracts of articles, and then the full-texts of articles to identify relevant SRs/MAs. In case of any disagreement, after asking the opinion of the third person, a decision was made based on majority agreement. Endnote version X9 Software was used to screen the articles.

### Data extraction

There were several reasons for selecting a non-Cochrane retracted systematic review article: First, according to the evidence, most Cochrane systematic review studies have been retracted due to updating-related issues, which are unrelated to error or scientific misconduct [21]. On the other hand, many of these articles do not receive the retraction tag in databases such as MEDLINE, so they cannot be identified as retracted publications [21]. Finally, since one of our objectives was to assess the methodological quality of the retracted systematic review articles, according to the evidence, non-Cochrane SRs tend to use less rigorous methods than Cochrane reviews [22]. The data related to the articles included in the present study were collected using a form designed in Excel 2019 software. Information related to the characteristics of the articles such as the title, year, and month of publication/retraction of the article and the country of the corresponding author was collected. The information related to the journal, such as the name of the journal and the impact factor of 2021, was collected according to the Web of Science (ISI).

There are different methods for classifying the reasons for the retraction. Considering that the degree of importance of scientific and non-scientific errors or misconduct is different, we used the method of reporting the reasons for retractions by Feng et al. [23]. Therefore, the reasons for retraction were classified into four groups: no scientific error and no academic misconduct, no scientific error and academic misconduct, scientific error and academic misconduct, and scientific error and no academic misconduct. Articles indexed in Retraction Watch always had more than one reason for retraction, and the most important reason for retraction was based on the placement of the article in one of the four groups. Due to the fact that some articles were not indexed in Retraction Watch, both the Retraction Watch and the retraction notice for each article were checked. More information about reasons for retraction is presented in Table 1. The data relating to the retraction requester/retractor and the retraction time were collected from the notice of the retraction in the PubMed database or the page of the journal that published the retracted article. It is necessary to mention that two researchers independently evaluated the articles in terms of grouping reasons for retraction and quality assessment. In case of any disagreement, a decision was made by referring to the full text of the article and discussing the reason for the disagreement between the three authors, and eventually, the final decision was made based on the consensus of the majority.

**Table 1:** Two-dimensional categorization for retraction reasons

Items	Academic misconduct	No academic misconduct
**Scientific errors**	Falsification/fabrication (data, image, results); manipulation (images, results); plagiarism (data, image); sabotage (materials, methods); fake peer review	Concerns/issues (data, image, results); contamination (cell lines/tissues, materials, reagents); error (analyses, cell lines/tissues, data, materials, methods, results/conclusions); results not reproducible; unreliable (data, image, results)
**No scientific errors**	Duplication (article, data, image, text); euphemisms (duplication, misconduct, plagiarism); forged authorship; misconduct (official investigation/finding, author, company/institution, third party); plagiarism (article, text)	Author unresponsive, breach of policy (author, third party); complaints (author, company/institution, third party); concerns/issues (authorship, referencing/attributions, third party involvement); conflict of interest; error (journal/publisher, third party, text)

Source: This table is taken from a part of the appendix of Feng et al.’s study [23]

### Study quality assessment

The authors used AMSTAR-2 to evaluate the quality of retracted systematic reviews. The AMSTAR-2 scale has the 16 items. Two reviewers (LK and RZ) answered questions with “yes” (score 1), “no” (score 0), or “partial yes” (score 0.5) in each article. Based on Li et al.’s study, critical domains in AMSTAR-2 were Items 2, 4, 7, 9, 11, 13, and 15, and the overall score in each SR/MA review was categorized into four classes including high articles assigned with *No* or one non-critical weakness, moderate articles specified with more than one non-critical weakness, and low article containing one critical flaw with or without non-critical weaknesses, and finally, critically low articles have more than one critical flaw with or without non-critical weaknesses*.* It should be mentioned that to investigate the relationship between the reasons for retraction and the methodological quality of retracted non-Cochrane SRs, the reason for retraction was not found in one systematic review study. Therefore, the evaluation was done on 281 articles.

### Data analysis

Firstly, Endnote version 9 was used to screen the data, and then, the data related to retraction and evaluation of methodology quality were collected in checklists designed in Excel 2019. Finally, data was analyzed in the Excel 2019 and SPSS 21 software. Descriptive statistics and chi-square and Fisher’s exact tests were used for analytical statistics.

## Results

### Search results

**Fig. 1:**
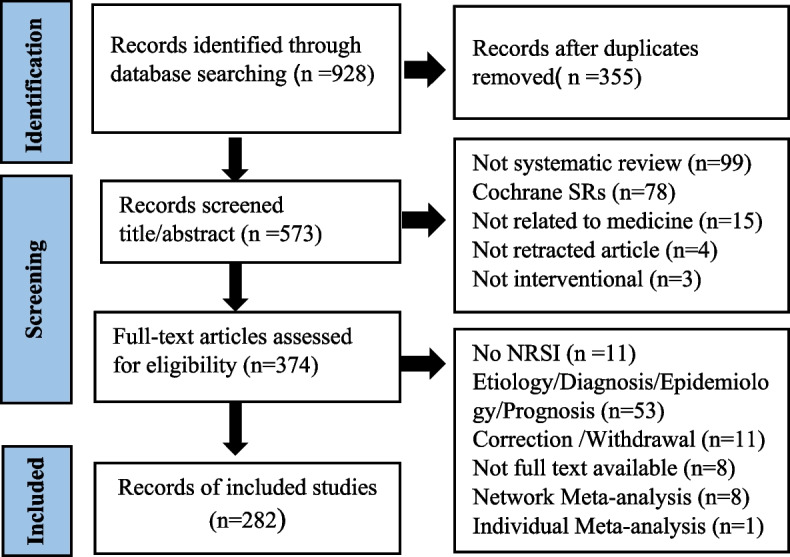
Study selection flow diagram

A total of 928 articles were extracted by searching three databases including WOS, Scopus, and PubMed. Three hundred fifty-five duplicate articles were excluded, and 291 articles were removed for reasons such as no systematic review, not related to medicine, and an etiology/diagnosis/epidemiology/prognosis systematic review. Finally, 282 retracted non-Cochrane SRs were included in this study. Details are provided in Fig. 1.

### General characteristics

#### Publication and retraction year

**Fig. 2:**
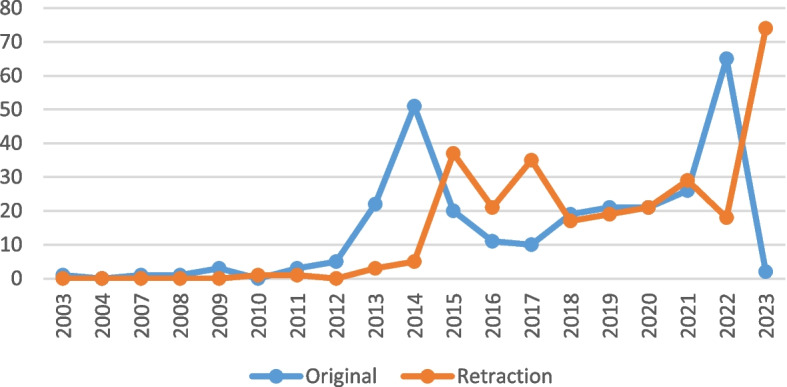
Publication and retraction year of retracted non-Cochrane SRs

The largest number of articles were published in 2022 (65 articles), while the highest number of retracted articles were related to the year 2023 (74 articles). In addition, about 142 articles (53.35%) have been retracted in the last 4 years. Details are reported in Fig. 2. The average interval between the time of publication and retraction was almost 23 months and 17 days. The shortest and longest time between publication and retraction of articles was 1 and 141 months respectively. More information is provided in Fig. 2.

#### Journals that published retracted articles

The findings showed that the journals published the most retracted SRs were *Tumor Biology* (26 articles) and *Computational and Mathematical Methods in Medicine* (24 articles). In addition, more than 86% of the retracted non-Cochrane SRs were published in journals with an impact factor above two. According to the affiliation of the corresponding authors, most of the retracted non-Cochrane SRs (about 73%) were from China. More information is provided in Table 2.

**Table 2:** Characteristics of the retracted non-Cochrane SRS

No	Journal name	No. of articles (%)
1	*Tumor Biology*	26 (9.2)
2	*Computational and Mathematical Methods in Medicine*	24 (8.5)
3	*Biomed Research International*	14 (4.96)
4	*Molecular Biology Reports*	14 (4.96)
5	*Medicine*	12(4.25)
6	*PloS One*	10(3.54)
7	*Journal of Healthcare Engineering*	8 (2.82)
8	*Contrast Media & Molecular Imaging*	6 (2.12)
9	*Frontiers in Surgery*	6 (2.12)
10	*European Journal of Medical Research*	5(1.77)
11	*Evidence-Based Complementary and Alternative Medicine*	5(1.77)
12	*Computational Intelligence and Neuroscience*	4 (1.41)
13	*Journal of Orthopedic Surgery and Research*	4(1.41)
14	*Disease Markers*	3 (1.06)
15	*Molecular Neurobiology*	3 (1.06)
16	Others	138 (48.93)
**Impact factor of journals**		**No (%)**
1	0 < IF ≤ 2	44 (15.60)
2	2 < IF ≤ 4	193 (68.43)
3	4 < IF ≤ 6	25 (8.86)
4	IF > 6	17 (6.02)
5	Not indexed in SCI	3 (1.06)
**Country of corresponding author**		**No (%)**
1	China	208 (73.75)
2	UK	10 (3.54)
3	USA	9 (3.19)
4	Japan	8 (2.82)
5	Iran	6 (2.12)
6	Australia	5 (1.77)
7	Others	36 (12.76)

#### Requestor/retractor of retraction

According to the published retraction notice, the requester or retractor about 30% of the retracted SRs were publishers and editors. The details are provided in Table 3.

**Table 3:** Requestor/retractor retraction for publications

NO	Requestor/retractor	No. of articles
1	Publisher and Editor	85(30.14)
2	Publisher	63 (22.34)
3	Editors	52 (18.43)
4	Authors	32 (11.34)
5	Authors and Editors	18 (6.38)
6	No mention	17(6.02)
7	Publisher, Editor, and Author	11 (3.9)
8	Others	4 (1.41)

#### Retraction reasons

**Fig. 3:**
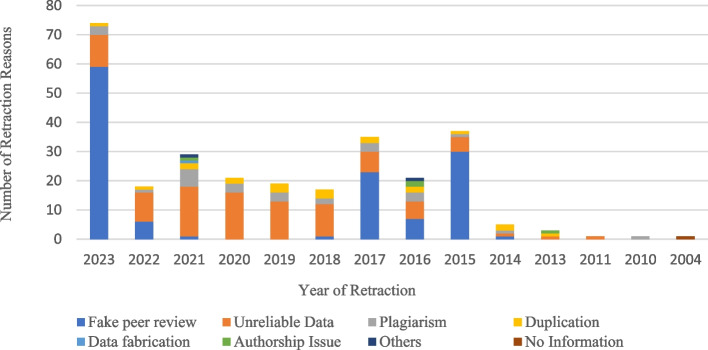
The number of retraction reasons based on the year of retraction

The findings showed that the most common reasons for retracting SRs/MAs articles were fake peer review (45.39%, 128 articles) which were retracted in 2023, 2015, and 2017 respectively. It is necessary to explain that two articles were retracted due to “Duplicate Publication by Journal/Publisher” and one article was retracted due to “Cites Retracted Work” being placed in the other group. The details are reported in Fig. 3.

### Methodological quality

#### Evaluation with AMSTAR-2 checklist

**Fig. 4:**
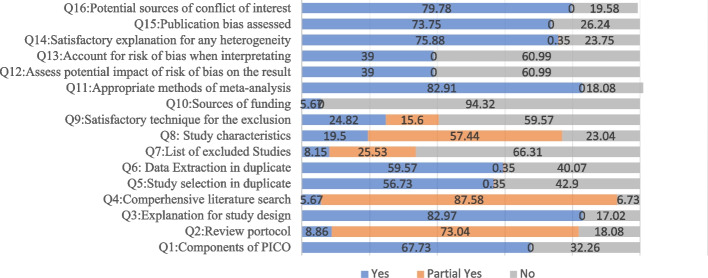
The evaluation of the quality of the methodology in the retracted non-Cochrane SRs with the AMSTAR-2 checklist

The results of the evaluation the methodology quality for the retracted non-Cochrane SRs with the AMSTAR-2 checklist showed that the highest amount of yes was related to question 3 (82.97%), question 11 (82.91%), and the highest amount of NO was assigned to the question 10 (94.32%), question 7 (66.31%), question 12 and 13 (60.99%), and question 9 (59.57%). Question 4 (87.58%), and question 2 (73.04%) had the highest amount of Partial Yes. The details are presented in Fig. 4.

#### Reasons for retraction and the methodological quality

In general, 202 articles (71.88%) had critically low quality, 51 articles (18.08%) low quality, 17 articles (6.02%) high quality, and 11 articles (3.90%) moderate quality. The results of Fisher’s exact test showed that there was a significant correlation between the reasons for retraction and the quality of the methodology (*P*-value < 0.05). Most of the articles that were critically low according to the AMSTAR-2 checklist were retracted due to scientific errors. The details are provided in Table 4.

**Table 4:** The relationship between the reason for retraction and the methodological quality of retracted non-Cochrane SRS

Reasons for retraction	Quality of methodology				Total	
	Critically low	Low	Moderate	High		
Scientific error and academic misconduct	100	26	1	4	131	*p* < 0.048
No scientific error and no academic misconduct	5	0	1	1	7	
No scientific error and academic misconduct	30	8	3	3	44	
Scientific error and no academic misconduct	67	17	6	9	99	
Total	202	51	11	17	281	

## Discussion

The results showed that about half of the non-Cochrane SRs were retracted in the last 4 years, and the average interval between the time of publication and retraction was about 23 months. A study by Kohl and Faggion Jr found that the retraction rate of medicine articles has increased in the last 5 years [24]. In Shi et al.’s study that reviewed non-Cochrane SRs [10] and Mena et al.’s study that analyzed urological articles [25], the average interval between publication and retraction of the article was about 20 months. The increase in the number of retracted articles in recent years and the short average interval between the time of publication and the retraction could be due to the use of plagiarism software to determine misconduct and the use of the COPE guidelines to assess the SRs/MAs [25]. Moreover, COPE in 2010 presented conditions for withdrawing articles and submitting a retraction statement. The special attention of the journals to this instruction could be one reason for the increase in the number of retracted articles in recent years.

The results showed that most of the non-Cochrane SRs have been published in journals with an impact factor greater than two, which was consistent with the study of Shi et al. [10]. In the study of Wiedermann [26], most of the retractions in the intensive care medicine fields were related to credible journals (2444). It can be concluded that the credible journals identified better misconduct and scientific errors [27]. On the other hand, publishing articles in credible journals leads to improving the professional and scientific status of researchers [28–30]; therefore, the possibility of fraud and misconduct in these journals is greater [27].

Moreover, the publisher and editor were the most retractors or requestors for retraction. In Shi et al.’s study, the most retractors were editors [10]. In Kardas et al.’s study, most of the requesters for retracting articles were editors and publishers [31]. In Soleimanpour and Panahi’s study, the most requesters who retracted the articles were the author and the editor, respectively [32]. Editors and publishers can help authors maintain their scientific and academic reputations by publishing a standard self-retraction notice for authors [33].

Based on the finding of this study, the majority of retracted SRs belonged to China, which findings were consistent with the studies of Chen et al., Wang et al., and Shi et al. [10, 15, 34]. Surveys showed that a large number of publications in scientific journals belong to Chinese authors [35]. Chinese researchers need to publish credible articles in order to be promoted and receive scientific awards, so these conditions could increase the risk of misconduct among these authors. On the other hand, the punishments by the Chinese organizations may be very mild in this regard [35].

In addition, most of the retracted SRs were due to reasons including fake peer reviews and unreliable data, respectively. In other studies, fake peer-review which is part of scientific errors and academic misconduct was the highest reason for retracting articles [10, 15]. In the study by Feng et al., which examined retracted articles in two journals, *Cell* and *Lancet*, 93% of the articles were also retracted due to scientific error [23]. Some authors, including non-English speakers or less experienced authors, use companies to publish their articles which may compromise the peer review process [36, 37]. In addition, the recommendations and selection of the reviewers based on the suggestions of the authors could lead to these types of errors [10, 38]. On the other hand, unreliable errors were another frequent scientific error in the reviewed articles. Considering that SR studies can provide stronger scientific evidence than primary studies, more attention should be paid to the use of standards in designing and conducting these studies. In addition, journals should not publish low-quality SRs [39, 40].

Based on the overall score of the AMSTAR-2 checklist, most of the articles were placed in the critically low category, as in the study of Leclercq et al., Storman et al., Almeida et al., and Matthias et al. [41–44]. Among the critical items of the checklist, items 7, 9, and 13 got the lowest scores which were consistent with the results of Kolaski et al., Min et al., Shang et al., Boini et al., and Li et al. [45–49], while item 11 received the most positive points. On the other hand, items 2 and 4 had the most partial yes score as in the Kamioka et al. study [50]. Indeed, assessing the risk of bias in individual studies prevents scientific errors such as Unreliable Data results, and errors in methodology and data falsification. Subsequently, the non-assessment of the risk of bias in individual studies makes their interpretation difficult, so the next critical item 13 did not get an acceptable score. By adhering to item 7, a clear picture of the excluded article is presented to reviewers and, consequently, the rate of retraction could be decreased. Duplication of the articles is related to critical item 4 in the AMSTAR-2 checklist and may happen for intentional or unintentional reasons. In case of unintentional error, it can be avoided by comprehensive search and choosing the proper keywords. Finally, registering the protocol of SRs/MAs is an important step to avoid errors.

## Conclusion

It is suggested that journals remove the proposed reviewer section for the SRs/MAs studies to prevent fake or fraudulent peer reviews. The order of the authors in SR/MA should not be important to encourage more teamwork. Moreover, according to the authors of this article, items 5 and 6 could also be considered critical items because they are a prelude to doing the next critical items correctly. The COPE guidelines registration protocol and checklists like AMSTAR-2 need to be observed while submitting SRs/MAs. Authors should improve their skills in methodology or use a medical librarian to select proper keywords, comprehensively search, and also get help from experts to perform statistical analysis including the risk of bias and heterogeneity. In order to comply with the ethical principles in writing these types of articles, more attention should be paid because these types of articles are used in evidence-based medicine and incorrect results may endanger people’s health.

## Limitation

This study faced several limitations. First, the protocol of this study was not registered. Second, for a limited number of articles in some databases, the retract tag was not used that was solved by searching three databases and checking the platforms of the journals that published these articles. Third, a small number of articles were excluded due to a lack of access to the full text as only the retraction notice was available. However, it was not possible to communicate with the authors of all the articles, while in the case of interaction with the authors, the answers to questions 7 and 10 might change.

### Supplementary Information

**Additional file 1.** Search strategy.

**Additional file 2.**

**Additional file 3.**
